# Deceased Kidney Donor Biomarkers: Relationship between Delayed Kidney Function and Graft Function Three Years after Transplantation

**DOI:** 10.3390/diagnostics14070717

**Published:** 2024-03-28

**Authors:** Rima Maslauskiene, Ruta Vaiciuniene, Peteris Tretjakovs, Gita Gersone, Aurelija Radzeviciene, Andrejus Bura, Edgaras Stankevicius, Inga Arune Bumblyte

**Affiliations:** 1 Department of Nephrology, Medical Academy, Lithuanian University of Health Sciences, LT-44307 Kaunas, Lithuania; ruta.vaiciuniene@lsmu.lt (R.V.); andrejus.bura@lsmu.lt (A.B.); ingaarune.bumblyte@lsmu.lt (I.A.B.); 2 Department of Human Physiology and Biochemistry, Riga Stradins University, Dzirciema Str. 16, LV-1007 Riga, Latvia; peteris.tretjakovs@rsu.lv (P.T.); gita.gersone@rsu.lv (G.G.); 3 Institute of Physiology and Pharmacology, Medical Academy, Lithuanian University of Health Sciences, A. Mickeviciaus Str. 9, LT-44307 Kaunas, Lithuania; aurelija.radzeviciene@lsmu.lt (A.R.); edgaras.stankevicius@lsmu.lt (E.S.)

**Keywords:** kidney transplantation, donor biomarker, NGAL, KIM-1, IL-18, CXCL10

## Abstract

With an increasing number of marginal donors, additional methods for the evaluation of cadaveric kidney quality are required. This study aimed to evaluate pretransplant deceased donor serum (s) and urine (u) biomarkers, including neutrophil gelatinase-associated lipocalin (NGAL), kidney injury molecule-1 (KIM-1), interleukin-18, and C-X-C motif chemokine 10 (CXCL10) for predicting early and late graft function. In total, 43 deceased kidney donors and 76 corresponding recipients were enrolled. Delayed graft function (DGF) occurred in 27.6% of cases. sIL-18, sKIM-1, uNGAL, and uKIM-1 were predictors of DGF. A model incorporating sIL-18, uKIM-1, and clinical factors was developed to predict DGF (AUROC 0.863). Univariate analysis showed a negative association between uKIM and graft eGFR at 6, 12, 24, and 36 months, but this was not confirmed in the multivariate analysis. In conclusion, we report a superior performance of donor biomarkers for predicting DGF and later graft function over serum creatinine. Higher levels of donor sIL-18 and uKIM in conjunction with expanded-criteria donors and longer cold ischemia times predicted DGF. With no renal tubular damage in zero-time donor biopsies, higher pretransplant urine and serum NGAL levels were associated with better allograft function one year after transplantation, and sNGAL with graft function three years after transplantation.

## 1. Introduction

Kidney transplantation represents the optimal choice for selected patients with end-stage renal disease. Compared to dialysis, transplantation improves patient survival and quality of life and reduces cardiovascular morbidity [1,2,3,4]. For kidney transplantation, the healthcare costs are significantly lower than long-term dialysis expenses [5,6,7]. The worldwide scarcity of donated kidneys has prompted various efforts to expand organ supply, such as accepting organs from older donors with acute kidney injury or different comorbidities [7]. Due to the increasing use of marginal organs, delayed graft function (DGF) is the most common early complication, ranging from 20.5 to 29.5% in deceased donors [8,9,10] and 45–65% in donation after circulatory death [11]. DGF is associated with prolonged hospitalization, increased mean treatment costs [12], and an increased risk of early infectious complications, mainly urinary tract infections and BK viremia. Prolonged DGF duration is also a risk factor for acute rejection [13,14,15]. Many studies have found that DGF is associated with worse graft later outcomes [13,14,16,17,18]. The main donor-related risk factors for DGF are ischemia–reperfusion injury, a deceased donor, advanced donor age, expanded-criteria donors (ECDs), donor acute kidney injury (AKI), higher body mass index, race, longer cold ischemia time, and recipient-related factors such as pretransplant dialysis, HLA mismatch, and other recipient clinical conditions [19]. Success in organ transplantation lies in avoiding DGF and prolonging graft survival.

The outcomes of transplantations using ECDs and donors with AKI remain a topical issue. Current clinical practice evaluates donor kidney suitability according to urine output and serum creatinine levels. Although serum creatinine (sCr) is typically used as a marker to monitor donor kidney function and diagnose AKI, it is an insufficiently sensitive and reliable biomarker of glomerular filtration, not a marker of tubular damage.

Furthermore, donor kidney injury can exist without any change in sCr. sCr concentration may not increase when half of the kidney function remains due to compensatory increases in other nephron functions [20]. The kinetics of the rise in sCr are relatively slow after acute injury [21].

Additional methods are required for the more accurate evaluation of donor kidney injury. In recent decades, great interest has been shown in finding non-invasive, reliable, and predictive biomarkers [22,23]. Some proteomics have been developed as biomarkers for kidney damage. Neutrophil gelatinase-associated lipocalin (NGAL), kidney injury molecule-1 (KIM-1), and interleukin-18, reflecting early pathological processes, have been studied in recent years, with varying results of clinical success. NGAL, KIM-1, and IL-18 have been shown to be markers for the early diagnosis of AKI or ischemic injury. In the last few years, several studies have indicated that C-X-C motif chemokine 10 (CXCL10) is involved in developing renal diseases. Increasing evidence suggests that CXCL10 plays an essential role in the inflammatory mechanisms induced by AKI. Also, various studies have established an association between CXCL10 levels and inflammatory/immune processes occurring during organ transplantation [24].

Our study aimed to evaluate potential biomarkers in pretransplant donors for predicting delayed graft function and graft outcomes over three years after kidney transplantation.

## 2. Materials and Methods

### 2.1. Study Design and Participants

This study is a joint project of the Lithuanian University of Health Sciences (Lithuania) and Riga Stradins University (Latvia). The prospective observational study enrolled kidney donors and corresponding recipients who underwent kidney transplantation between May 2017 and October 2020 in the Kaunas Clinics of the Lithuanian University of Health Sciences. Exclusion criteria were as follows: participants (donors and recipients) under the age of 18 years, primary recipient graft failure due to surgical causes, repeated transplants when immunosuppressive therapy was still being used, refusal of the recipient or legal representative of the donor to participate in the study. 

The study enrolled 43 deceased donors and 76 kidney recipients. All donors included in the study were prepared in one university hospital following the same standards of diagnosis and treatment. All kidney transplant donors and recipients were of Caucasian descent. Expanded-criteria donors (ECDs) were defined according to the United Network for Organ Sharing (UNOS) criteria: kidney donors over the age of 60 years without comorbidities or donors over the age of 50 years with two comorbidities (history of hypertension, death from cerebrovascular accident, or terminal serum creatinine levels > 132 µmol/L) [25]. Delayed graft function (DGF) was defined by dialysis requirement during the first week after transplantation, as defined by UNOS [26]. To identify recipient-related factors for DGF, we compared several of the essential characteristics of recipients divided into immediate graft function (IGF) and DGF groups. IGF was established as patients without a need for dialysis during the first seven days after transplantation. 

For the biomarker investigation, donors’ serum and urine samples were collected before kidney explantation on the day of procurement. Donor kidney function was evaluated on the day of organ donation by measuring the creatinine concentration in serum. Serum creatinine concentration was measured by the kinetic Jaffe (compensated) method, traceable to the IDMS (isotope dilution mass spectrometry) reference method (Analyzer AU680, Beckman Coulter, Chaska, MN, USA). The estimated glomerular filtration rate (eGFR) was calculated using the CKD-EPI (Chronic Kidney Disease Epidemiology Collaboration (2012)) equation. 

All clinical data were obtained from medical records. Several commercially available biomarkers for kidney injury were tested in both serum and urine, including neutrophil gelatinase-associated lipocalin (NGAL), interleukin-18 (IL-18), and kidney injury molecule-1 (KIM-1). C-X-C motif chemokine 10 (CXCL10) was tested in serum. Recipient follow-up data were collected for three years after kidney transplantation. Renal function was evaluated 6, 12, 24, and 36 months after transplantation. The observation period ended early in cases of graft loss, death, or the date of the last documented clinical contact with the transplant recipient. Routine laboratory values were analyzed in the hospital’s local laboratory as part of routine follow-up care. 

A time-zero graft biopsy was performed by the urologist in the kidney transplant operating room during surgery when kidney blood flow was restored. The histological findings in the kidney biopsies were evaluated by pathologists at the National Center of Pathology based on the Banff criteria [27]. Acute rejection episodes were defined as either biopsy-proven or clinically suspected acute rejection improved by empirical steroid pulse therapy.

The Kidney Donor Profile Index (KDPI) was included for additional information about the donors in this study. This index is not routinely used in our transplantation center. The KDRI was calculated retrospectively using ten donor characteristics (age, height, weight, ethnicity, hypertension, diabetes, cause of death, serum creatinine, hepatitis C virus status, and information about meeting donation after cardiac death criteria) determined by the Organ Procurement and Transplantation Network calculator (Available online: https://optn.transplant.hrsa.gov/resources/allocation-calculators/kdpi-calculator, accessed on 28 January 2024). Based on the KDRI values, each kidney’s KDPI score (%) was determined using the Organ Procurement and Transplantation Network (OPTN) mapping table and the donor median for the corresponding year. 

The primary outcome was determining donor biomarkers’ predictive performance for DGF, and the secondary outcome was establishing donor biomarkers’ relationships with 6-month, 1-, 2-, and 3-year graft function. 

### 2.2. Biomarker Measurement

Per study protocol, blood and urine samples were obtained from donors before organ procurement on the day of organ explantation. Fresh urine samples were collected using an indwelling urinary catheter tube and centrifuged for 10 min at 2500× *g* to remove insoluble elements. Supernatants were divided into 1 mL aliquots and stored at −80 °C. 

Donor blood samples were left for 30–60 min at room temperature for a clot to form, then were centrifuged, and the supernatant was aliquoted for freezing at −80 °C. Donors’ serum and urine samples were transported to Rigas Stradins University Laboratory, ensuring the necessary temperature conditions. The same experienced specialists performed the biomarker tests. They were blinded to clinical information about the participants. Samples were thawed at 37 °C before analysis. After one freeze–thaw cycle, serum and urine biomarkers were measured as recommended by the manufacturer.

Serum and urine NGAL measurement was performed using a commercial enzyme-linked immunosorbent assay (ELISA) kit (Human Lipocalin-2/NGAL ELISA Kit; Sigma-Aldrich Chemie GmbH, Taufkirchen, Germany). The serum and urine KIM-1 assays were performed using a Quantikine ELISA kit (R&D Systems Europe, Ltd., Abingdon, UK), the serum and urine IL-18 assays were measured using an ELISA kit (Human IL18/Interleukin-18 ELISA Kit; Sigma-Aldrich Chemie GmbH), and the serum and urine IP-10/CXCL10 assays using an ELISA kit (Sigma-Aldrich Chemie GmbH). Biomarkers were expressed without correction for creatinine levels.

### 2.3. Statistical Analysis

Descriptive statistics are reported as mean (SD) for data with normal distribution, median (interquartile range or minimum and maximum values) for continuous variables with non-normal distribution, and frequency (percentage) for categorical variables. Chi-squared tests were used for the categorical variables. Continuous variables were compared using Students’ *t*-tests. Mann–Whitney *U*-tests and Kruskal–Wallis tests were used to measure differences in continuous variables with a skewed distribution. Correlations between urine biomarkers and continuous variables were assessed by Spearman or Pearson correlation, depending on the distribution of variables. The diagnostic performances of biomarkers to predict DGF were evaluated by receiver-operating characteristic (ROC) curve analyses, and the cut-off point for DGF occurrence was determined by the maximum values of the Youden-J indexes. Factors significantly different between the DGF and IGF groups in the univariate analyses were included in multivariate logistics analyses. Non-normally distributed biomarker values were log_10_-transformed. Multiple logistic regression analyses with a backward variable selection were used to assess the association between biomarkers and DGF in the presence of covariates and to produce the best-fit model for predicting DGF. The results are presented as odds ratios (OR) with a 95% confidence interval [95% CI] and the *p*-value of the likelihood-ratio test. The diagnostic efficacy of the best-fit model for DGF progression was evaluated through ROC curve analysis utilizing the DeLong test. Univariate linear regression was used to estimate the impact of donor and recipient variables on eGFR at 6-month, 1-year, 2-year, and 3-year intervals. Multivariable linear regression with a stepwise variable selection procedure was conducted in the univariate analysis to identify independent factors influencing graft function. The results are presented as regression coefficients (B) with 95% confidence intervals. All tests of significance were two-sided, with *p* < 0.05 considered significant. Data were analyzed using IBM SPSS Statistics Version 29.0 (New York, NY, USA) and MedCalc (Statistical Software Ltd., version 22.016, Ostend, Belgium).

## 3. Results

### 3.1. Characteristics of the Study Population

The prospective observational study enrolled 43 deceased donors. More than half of the donors (53.5%) met the ECD criteria. In total, 79.1% of the donors required inotropes to maintain hemodynamic stability. The mean age of the donors was 53.51 (SD 13.33) years (20–74 years); 37.2% of the donors were older than 60 (respectively, 52.4% in the DGF group and 21.8% in the IFG group). There were 25 (58%) men and 18 (41.9%) women. The mean KDRI was 1.1686 (SD 0.358), the mean KDPI was 59.14% (SD 26.57), and 18.5% had a KDPI of more than 80%. The most common death cause was a cerebral vascular accident (72.1%). The number of deaths from head trauma and other causes was equal [14%]. The study involved one donor after circulatory death, and 58.1% of donors had a history of hypertension. The average donor’s body mass index was 25.87 (SD 3.62). On the day of kidney donation, creatinine ranged from 22 µmol/L to 221 µmol/L, with an average of 95.13 (SD 39.35) µmol/L. The median urine output was 3330 (2400–4000) mL/24 h. The creatinine level was elevated over 26.5 µmol/L (≥0.3 mg/dL) above normal, which could be considered AKI in 16.7% of women and 12.0% of men. Time-zero graft biopsies were performed in 75 cases. There was no significant tubular damage, such as acute tubular injury or acute tubular necrosis. The values of the donor biomarker concentrations are given in Supplementary Table S1. 

The study involved 76 kidney recipients. The recipients’ characteristics and transplantation details are summarized in Table 1. 

Out of the total number of kidney transplantations performed, four [5.3%] were pre-emptive. Three (3.9%) patients were on peritoneal dialysis. None of these before-mentioned patients experienced DGF. Of the recipients, 90.8% underwent their first kidney transplantation, while 9.2% had repeated transplantations. Of them, 5.3% underwent a second transplantation, 2.6% had a third, and 1.3% had a fourth transplantation. The median waiting time for kidney transplantation was 228 (13–1332) days. The most common cause of end-stage renal disease was chronic glomerulonephritis (31.6%), followed by polycystic kidney disease at 13.2%, diabetes at 9.2%, pyelonephritis at 9.2%, arterial hypertension at 5.3%, and other reasons at 31.6%. There were no significant differences between the DGF and IGF groups according to kidney disease diagnosis. Antibody induction was given to 65 (85.5%) recipients. The immunosuppressive protocol was triple therapy for all patients with methylprednisolone, mycophenolate mofetil, and a calcineurin inhibitor (cyclosporine or tacrolimus). DGF occurred in 27.6% of recipients with a need for 1 to 16 sessions of hemodialysis. 

Recipients who had worse early graft function had significantly longer dialysis vintage. Cold ischemic time was longer in the DGF group. Patients with DGF had more extended hospital stays (Table 1).

Donors in the DGF group were older than those in the IGF group and had a more significant ECD ratio (76.2 vs. 41.8%), with a higher KDPI percentage (45 vs. 13%). Donor creatinine was not significantly different between the groups (*p* = 0.511) (Table 2).

The serum NGAL (sNGAL), urinary IL-18 (uIL-18), and serum CXCL10 values did not differ between the DGF and IGF groups. Other biomarkers such as serum IL-18 (sIL-18), serum KIM-1, urinary NGAL (uNGAL), and urinary KIM-1 (uKIM-1) levels were significantly higher in the DGF group compared to the IGF group (Table 3). There was a statistically significant correlation between the donors’ serum creatinine and the donors’ sKIM-1 (r = 0.334, *p* = 0.04), sNGAL (r = 0.360, *p* = 0.002), and uNGAL (r = 0.2900, *p* = 0.037). 

During follow-up, recipients with DGF showed significantly lower kidney function than those without DGF after 1, 3, 6, 12, and 36 months (Supplementary Table S2). No significant differences in graft function after 24 months were observed between the DGF and IGF groups. Acute rejection occurred in 10.5% (*n* = 8) of recipients during the first year, with a significant difference between the DGF (23.8%) and IGF (5.5%) groups (*p* = 0.033). No statistically significant differences existed between donor biomarker concentrations and acute rejection during the first year after transplantation. 

### 3.2. Predictive Performances of Donor Biomarkers for Delayed Graft Function

The diagnostic performances of biomarkers for predicting DGF were assessed using ROC curve analysis. sIL-18, sKIM-1, uNGAL, and uKIM-1 were found to be significant predictors of DGF, whereas serum NGAL (*p* = 0.150), urine IL-18 (*p* = 0.222), and sCXCL10 (*p* = 0.648) were not. The diagnostic performances of the biomarkers’ area under the receiver-operating characteristic curve (AUROC) with optimal cut-off values for the risk of DGF are shown in Table 4. 

We developed a model to evaluate the performance of donor biomarkers in predicting DGF. The model incorporates the donor biomarkers and factors identified in previous clinical studies that are known predictors of DGF [28]. Univariate logistic regression analyses showed that donor age, ECD, KDPI, cold ischemia time, and serum and urine KIM-1 and sIL-18 were significantly associated with DGF. Surprisingly, there was not statistically significant (*p* = 0.087) association between uNGAL and DGF (Table 5).

The multivariate logistic regression analysis demonstrated that the reduced best-fit model, which may be helpful to predict graft function in the early period after transplantation, included donor evaluation (extended or standard criteria donor), cold ischemia time, and two biomarkers—sIL-18 and uKIM-1. However, variables significant in the univariate analysis, such as donor age, KDPI, higher than eighty percent KDPI, and sKIM-1 concentration, were insignificant in the multivariate analysis (Table 5). 

**Figure 1:**
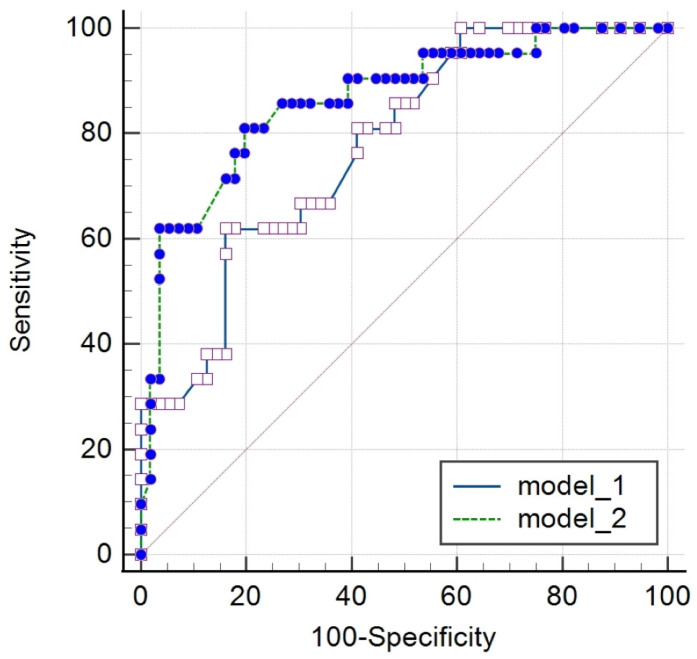
Comparison of diagnostic accuracy of the receiver-operating characteristic curves of the reduced best-fit models with and without biomarkers. Model 1—donor evaluation (ECD or SCD) and cold ischemia time. Model 2—donor evaluation (ECD or SCD), cold ischemia time, and biomarkers uKIM-1 and sIL-18. Difference between areas = 0.0872; *p* = 0.0324 in DeLong test [95% CI 0.00731–0.167]. CI—confidence interval; ECD—extended-criteria donors; SCD—standard-criteria donors; uKIM-1—urine kidney injury molecule-1; sIL-18—serum interleukin-18.

Using predictors associated with DGF, as indicated by the multivariate logistic regression analysis, we performed an ROC curve analysis to assess the diagnostic performance of the reduced best-fit model. The AUROC of the model was 0.863 [95% CI 0.651–0.856], *p* = 0.0006. This model included biomarkers (uKIM-1, sIL-18) and selected clinical data concerning donor evaluation type (ECD or SCD) and cold ischemia time. Analogously, a reduced best-fit model without biomarkers was performed based only on the same clinical data. The AUROC of this model was 0.765 [95% CI 0.766–0.930], *p* < 0.0001, and it had a significantly lower ability to predict DGF versus the biomarkers-included model (*p* = 0.0324; DeLong test) (Figure 1). 

### 3.3. Donor Biomarker Associations with Later Graft Function

We did not find a significant correlation between donor creatinine and recipient graft function up to three years after transplantation. Only uKIM showed a statistically significant negative correlation with graft function throughout the observation period at 1 month (r = −0.415, *p* = 0.03), 3 months (r = −0.359, *p* = 0.01), 6 months (r = −0.361, *p* = 0.009, 1 year (r = −0.329, *p* = 0.026), 2 years (r = −0.358, *p* = 0.02), and 3 years (r = −0.457, *p* = 0.004) after transplantation. NGAL did not correlate with eGFR in the early post-transplant period. However, a correlation was found with sNGAL and eGFR at six months (r = 0.251, *p* = 0.063), 1 year (r = 0.263, *p* = 0.037), 2 years (r = 0.369, *p* = 0.004), and 3 years (r = 0.329, *p* = 0.017). uNGAL correlated with kidney function only after one year (r = 0.308, *p* = 0.045). 

Univariate linear regression analysis showed a significant negative association between uKIM and renal function at six months (β coefficient = −0.361, [95% CI −48.11 to −37.129], *p* = 0.009), but this was not confirmed in the multivariate analysis. 

Univariate linear regression analyses found that donor age, KDPI, cold ischemia time, DGF, uKIM-1, serum, and urine NGAL were significantly associated with 1-year eGFR. Multiple linear regression analyses with a stepwise variable selection showed that donor age, cold ischemia time, and uNGAL are significant predictors of 1-year graft function after adjusting other variables (Table 6). KDPI and donor age correlated; therefore, we chose to analyze age because KDPI is not routinely used in our center.

In the univariate linear regression analyses, sNGAL and uKIM-1 were significantly associated with 2-year eGFR (sNGAL β coefficient = 0.349, *p* = 0.004; uKIM-1 β coefficient = −0.358, *p* = 0.020), but their significance was not confirmed in the multivariate analysis. 

In the univariate linear regression analyses, sNGAL (β coefficient = 0.346, [95% CI 3.937–31.572], *p* = 0.013) and uKIM-1 (β coefficient = −0.457, [95% CI −51.806 to −10.685], *p* = 0.004) were significantly associated with 3-year graft function. In the multivariate linear regression analyses, sNGA, donor age, and cold ischemia time were predictors of 3-year eGFR (β coefficient = 0.289, [95% CI 0.334–30.84], *p* = 0.045) after the DGF and biomarkers were adjusted. 

## 4. Discussion

This observational study aimed to evaluate clinically available and relatively simple tested donor biomarkers to predict delayed graft function and later outcomes up to three years after transplantation. 

The routine testing of donor sCr did not accurately predict early or late graft outcomes. We found that pretransplant uKIM-1, sKIM-1, sIL-18, and uNGAL were reliable for predicting the occurrence of DGF. We determined the cut-off values of biomarkers and developed a reduced best-fit model that could be helpful in predicting graft function in the early post-transplant period in clinical practice. After adjusting for other variables, the model revealed the prognostic value of donor evaluation (extended or standard criteria donors), cold ischemia time, and two biomarkers—sIL-18 and uKIM-1. The diagnostic performance of this model was found to be significantly better than that of the donor clinical factors alone. The predictive performance of the donor biomarkers for later graft function was also tested. Donor biomarkers were significantly associated with 1-year graft outcomes (uKIM-1 negatively; uNGAL and sNGAL positively), but ultimately, only uNGAL’s predictive value with donor age was confirmed in the multivariate analysis. Three-year outcomes were associated with pretransplant sNGAL. 

The evaluation of kidney graft quality is critical to effectively increase the donor pool and improve short- and long-term transplant outcomes. Grafts from ECDs yield inferior outcomes than standard-criteria donors, with higher incidences of DGF and primary non-function. Finding additional methods to evaluate the deceased donor’s kidney quality is crucial in this field. 

In the last few years, many new technologies have emerged to examine organ function, including transcriptomics, genomics, proteomics, metabolomics, and innovative solutions in organ perfusion [29]. Numerous potential biomarkers, quantified in serum, urine, renal tissues, and perfusate, have been tested to diagnose donor AKI and predict DGF, acute rejection, and chronic allograft dysfunction. Most published studies analyze recipients’ materials, but fewer significant studies analyzing donor biomarkers have been performed.

In this study, particular attention was drawn to donor biomarkers. Our results showed that donor sCr levels did not predict early or later graft outcomes. These findings are consistent with the existing literature showing that donor AKI diagnosis based on sCr is not associated with worse recipient outcomes in the long term [30,31], while other authors find generally less favorable graft outcomes if the donor had an AKI [32,33]. 

In this study, sKIM-1 correlated with donor sCr, while uKIM-1 was associated with DGF. This is a novel finding which needs to be confirmed in further studies. Kidney injury molecule-1 is expressed in the kidney proximal tubular cells, liver, and spleen. uKIM-1 is recognized as an early and specific urinary biomarker for kidney injury and has been tested in many AKI studies [34]. There are a small number of studies that have tested blood KIM-1 in kidney disease [35,36]. Recently, a small study analyzed the diagnostic performance of serum and urine KIM-1 in renal donors and recipients to predict DGF. However, the authors did not produce statistically significant results [37]. Field’s published study on multi-organ donors found that a higher donor urinary KIM -1 level was associated with a worse early function of transplanted kidneys [38]. P. Reese et al., in a large prospective study, found that even though the levels of tested donor biomarkers were higher in recipients with DGF, the association of uKIM-1 with DGF was significant only for the middle biomarker tertiles [39].

Another donor biomarker associated with DGF in this study was uNGAL. NGAL is primarily known as a biomarker of acute kidney injury and is released after tubular damage and during the processes of renal regeneration. NGAL may play a role as a predictor of renal function decline and mortality due to kidney failure [40]. We confirmed the results published by Halmen et al., showing that donor NGAL concentrations correlated directly with donor plasma creatinine levels. In the study by Halmen et al., high donor uNGAL concentration was associated with prolonged DGF and more histological changes in the donor kidney biopsies. Still, uNGAL and sNGAL failed to predict DGF. It is important to note that this study did not include donors with AKI based on plasma creatinine levels [41]. P. Reese et al. found that the levels of donor uNGAL were higher in recipients with DGF. However, the multivariate analysis revealed a positive association between donor uNGAL concentrations and a modest increase in the relative risk of recipient DGF [39]. Koo et al. investigated the levels of donor urine biomarkers to predict reduced graft function and slow graft function. Multivariate analyses, adjusted for several donor factors, showed that donor uNGAL was associated with donor AKI and predicted reduced recipient kidney function [42]. Whether urine NGAL or plasma NGAL is the better predictor for graft outcomes remains an unresolved question among kidney recipients. A recent meta-analysis suggests that the recipient’s urine and serum/plasma NGAL are valuable biomarkers for the early identification of DGF in kidney transplantation [43]. The prognostic significance of donor sNGAL for graft outcomes has been little studied. Buemi et al. found no correlation between donor plasma NGAL and uNGAL values and the occurrence of DGF [44]. In our study, sNGAL was not associated with DGF, but had a prognostic value in later outcomes. 

Pretransplant IL-18 for the prediction of DGF was also examined. IL-18 is produced by T cells and macrophages and is a multifunctional cytokine involved in both mediating and predicting AKI. It mediates various inflammatory and oxidative responses, including renal injury, fibrosis, and graft rejection [45]. 

Studies have shown that IL-18 levels are significantly higher in the urine of patients with acute tubular necrosis and DGF [46]. Higher levels of urinary IL-18 in deceased donor kidney recipients within the first 24 h after transplantation have been associated with higher rates of DGF and suboptimal allograft function. However, the role of donor serum IL-18 as a biomarker to predict early graft dysfunction after kidney transplantation is poorly defined. Our study found that donor sIL-18 has a predictive value for DGF, but there are no correlations with later graft function.

In the last few years, some new biomarkers have been developed. Different studies have indicated that C-X-C motif chemokine 10 (CXCL10) is involved in developing renal diseases through the chemoattraction of inflammatory cells and facilitating cell growth and angiostatic effects [24]. This chemokine has been well studied as a graft-rejection predictor; its role in ischemic reperfusion injury has been shown in mice models [24,47]. Several studies have demonstrated that urinary CXCL10 expression is significantly elevated during AKI [48]. The pretransplant elevation of serum CXCL10 concentration in patients with acute rejection shows an association with the risk of graft failure [48,49]. Urinary CXCL10 levels increase in patients experiencing acute rejection [50] and in pediatric patients with declining renal allograft function [51]. A few studies evaluate donor CXCL10 as a biomarker and its value for the recipient’s graft outcomes. A recent multicenter study of 1100 deceased donors and 2869 recipients who underwent various types of transplants [1470 kidney recipients] found that the instability of hemodynamics, anoxia as a death cause, the presence of risk factors associated with cardiovascular disease, and the presence of active infection were significant predictors of donor serum CXCL10 levels. High CXCL10 level was significantly associated with a lower probability of immediate kidney graft function and predicted recipient survival after kidney, liver, and heart transplantation [52]. In our study, the level of serum CXCL10 was higher in the ECD group (*p* < 0.001) but was not associated with DGF or kidney transplant function in the three years after transplantation. 

The purpose of studies on biomarkers is to facilitate an early and personalized diagnosis and prognosis for each patient. A reduced best-fitting model, which included two selected donor biomarkers (sIL-18 and uKIM-1) and two critical clinical factors [donor evaluation—ECD or SCD—and cold ischemia time] was used in the logistic regression analysis in this study. The model may be helpful in deciding on medical intervention after transplantation. Additionally, cut-offs for medical decision making based on the predictive values obtained through data analysis are proposed. Koo et al.’s study also created a prediction model of early graft dysfunction based on donor biomarkers. This model included donor uNGAL, uL-FABP, and sCR, and had a better predictive value for reduced graft function than donor sCr alone [42]. This model may help evaluate the suitability of a potential donor kidney for transplantation. 

We evaluated donor biomarkers’ predictive performance for later graft function. 

uKIM-1 (negatively) and uNGAL (positively) were associated with 1-year graft outcomes, but only uNGAL predictive values were confirmed in the multivariate analysis. This is a different result from the study by Halmen et al., where high donor uNGAL concentrations were associated with worse 1-year kidney graft survival [41]. In Koo et al.’s study, uNGAL level negatively correlated with kidney function at 3, 6, and 12 months after transplantation. Urinary KIM and NGAL were negatively associated with 1-year eGFR in the univariate analyses, but their significance was not confirmed in the multivariate analysis. In another study, Modelina et al. found no statistically significant associations between donor urinary NGAL, KIM-1, or IL-18 concentrations above the cut-off levels and the 6-month graft eGFR [53]. In the study by Reese et al., pretransplant uNGAL was associated with 6-month eGFR only among recipients without DGF, whereas KIM-1 and IL-18 were not associated at all. The results of this study suggest that donor urinary biomarkers provide limited value in predicting recipient allograft function at six months post-transplantation [39]. The authors continued follow-up and concluded that the risk of graft failure and the 3-year composite outcome did not vary with donor injury biomarker concentrations after adjusting for donor, transplant, and recipient characteristics. Also, subclinical donor AKI (elevated urine injury biomarkers and normal sCr) was not associated with graft failure [54]. Various research findings suggest that further in-depth studies should be performed.

The role of donor serum biomarkers in predicting subsequent graft outcomes is poorly defined. Brain death causes a cytokine storm and inflammatory response that might explain the high levels of sNGAL in donors whose kidneys appear to be healthy [41]. In our study, only sNGAL had a prognostic value for later outcomes: sNGAL was significantly positively correlated with kidney function at 6, 12, 24, and 36 months. We found that sNGAL, along with other clinical data, may predict kidney function three years after transplantation. Based on evidence from other studies, we hypothesize that NGAL is an acute-phase protein involved in inflammation. One source of NGAL in serum is the activation process of neutrophils and monocytes during the acute phase of the reaction, so serum NGAL elevation may not result only from decreased kidney glomerular filtration or tubular damage. Its main antibacterial mechanism is the regulation of iron metabolism, but it may also influence chemotaxis, adhesion, and the migration of inflammatory cells. This suggests that NGAL may provide protection in AKI [40,55] and induce protective processes that support long-term recovery. We hypothesize that in the absence of donor chronic kidney disease and no signs of renal tubular damage in zero-time biopsies, a higher NGAL concentration also indicates a possible increase in renal reserve and graft function in the future.

Our study has several strengths. It is a prospective study with an extensive follow-up period of three years. While the surge in innovative studies is noteworthy, most biomarker measurement techniques require sophisticated technological apparatus and an extended duration to yield results. Our investigation incorporated an analysis of a panel of biomarkers, which included relatively straightforward tests for NGAL, KIM-1, IL-18, and the novel addition to the DGF field, CXCL10. Rapid biomarker tests would provide additional information about the status of the donor’s kidney and could personalize medical decisions after transplantation.

There are several limitations to our study. The small sample size limited the statistical accuracy, making it difficult to draw more conclusive predictions. The donors’ urinary biomarker values were not normalized to the biomarker/creatinine ratio; nevertheless, only a small number of donors in this study were polyuric. More extensive studies should be performed to confirm donor biomarkers’ relationships with kidney graft function. The follow-up of our study included the COVID-19 pandemic period, and some of the recipients experienced adverse events of this disease with some influence on graft function, independent of the quality of the donor’s kidney. 

## 5. Conclusions

We found that the level of biomarkers is a better predictor of DGF and later graft function compared to serum creatinine measured at the same time. Higher levels of donor serum IL-18 and urine KIM in conjunction with expanded-criteria donors and longer cold ischemia times predicted DGF better than clinical criteria alone. Without renal tubular damage in zero-time donor biopsies, higher pretransplant donor urine and serum NGAL were associated with better recipient allograft function one year after transplantation, and serum NGAL was associated with graft function three years after transplantation.

## Figures and Tables

**Table 1:** Characteristics of kidney transplant recipients, stratified by delayed graft function.

Clinical Characteristics	Total = 76	IGF *n* = 55 (73.4%)	DGF *n* = 21 (27.6%)	*p* Value
Mean age *, years	46.92 (13.39)	47.47 (12.85)	45.48 (14.94)	0.565
Female *n* (%)/male *n* (%)	29 (38.2)/47 (61.8)	23 (41.8)/32 (58.2)	6 (28.6)/15 (71.4)	0.288
Body mass index *, kg/m^2^	25.13 (4.71)	25.08 (4.66)	25.24 (4.94)	0.898
Duration of dialysis, month	22 (1–171)	20 (1–171)	27 (9–82)	**0.018**
Waiting on the recipient list, days	228 (13–1332)	224 (13–1332)	282 (69–1175)	0.128
Mode of dialysis HD, *n* (%)	69 (90.8)	48 (87.3)	21 (100)	0.229
First transplantation (%)	69 (90.8)	51 (92.7)	18 (85.7)	0.344
Time ischemia cold *, hours	15.63 (3.95)	14.81 (3.34)	17.73 (4.68)	**0.014**
Time ischemia warm, min.	35 (20–80)	35.0 (23–80)	36 (20–60)	0.686
HLA mismatch level, *n* (%)				0.597
0–3	48 (63.2)	36 (65.5)	12 (57.1)	
3–5	28 (36.8)	19 (34.5)	9 (42.9)	
Induction with antilymphocyte preparations, *n* (%)				0.741
Not intended	11 (14.5)	9 (16.4)	2 (9.5)	
Basiliximab	62 (81.6)	44 (80.0)	18 (85.7)	
ATG	3 (3.9)	2 (3.6)	1 (4.8)	
CNI				0.371
Cyclosporin, *n* (%)	16 (21.1)	13 (23.6)	3 (14.3)	
Tacrolimus, *n* (%)	60 (78.9)	42 (76.4)	18 (85.7)	
Lengths of stay in hospital *, days	19.9 (7.99)	18 (5.3)	25.2 (11.3)	**<0.001**

HD—hemodialysis; ATG—antithymocyte globulin; CNI—calcineurin inhibitor; DGF—delayed graft function; eGFR—estimated glomerular filtration rate; IGF—immediate graft function; PD—peritoneal dialysis. Values reported are median (min.–max.), means (SD *), or *n* (%). Continuous variables were compared using Mann–Whitney *U*-tests or Students’ *t*-tests as appropriate, and categorical variables were compared using the Chi-squared test. Bold formatting indicates statistically significant *p* values (*p* < 0.05).

**Table 2:** Kidney donor characteristics, stratified by delayed graft function.

Characteristics	IGF *n* = 55	DGF = 21	*p* Value
Donor age, years	49.65 (14.4)	59.9 (7.0)	0.003
Donor gender male/female (%)	54.5/45.5	57.1/42.9	0.839
Donor BMI, kg/m^2^	25.2 (3.5)	26.97 (3.5)	0.66
Cerebrovascular death cause (%)	70.9	76.2	0.778
History of hypertension (yes vs. no) (%)	50.9/49.1	66.7/33.3	0.303
Expanded-criteria donors, (yes vs. no) (%)	41.8/58.2	76.2/23.8	0.007
KDPI (%)	50.36 (26.6)	71.85 (19.8)	0.02
KDPI > 80 (%), (yes vs. no)	13/87	45/55	0.018
Donor urine output, mL/kg/h	1.85 (1.2–2.3)	1.51 (1.1–2.3)	0.41

BMI—body mass index; eGFR—estimated glomerular filtration rate; DGF—delayed graft function; IGF—immediate graft function; KDPI—kidney donor profile index. Values reported are presented as median (interquartile ranges), means (SD), or *n* (%). Continuous variables were compared using Mann–Whitney *U*-tests, and categorical variables were compared using the Chi-squared test.

**Table 3:** Comparison of donor biomarkers between immediate and delayed graft function groups.

Characteristics	IGF *n* = 55	DGF = 21	*p* Value
Donor serum creatinine, µcmol/L	89.6 (32.1)	99.4 (42.2)	0.511
Serum NGAL pg/mL	25,496.1 (13,583.0–42,939.6)	44,460.5 (15,664.3–62,027)	0.140
Serum IL-18 pg/mL	162.97 (108.18–243.3)	298.1 (160.6–380.5)	0.006
Serum KIM-1 ng/mL	224.8 (111.5–352.7)	283.0 (223.6–827.8)	0.010
Serum CXCL10 pg/mL	39.61 (29–98,9)	41.49 (29.0–134.5)	0.614
Urinary NGAL pg/mL	1219.1 (821.9–1219.1)	2921.7 (1208.4–5766.4)	0.047
Urinary IL-18 pg/mL	9.7 (3.3–17.6)	14.3 (6.0–23.4)	0.25
Urinary KIM-1, ng/mL	1.4 (0.7–3.97)	3.2 (2.4–6.5)	0.014

CXCL10—C-X-C-motif chemokine 10; DGF—delayed graft function; IGF—immediate graft function; IL-18—interleukin-18 (serum, urine); NGAL—neutrophil gelatinase-associated lipocalin; KIM-1—kidney injury molecule-1 (serum, urine). Values are presented as median (interquartile ranges), means (SD), or *n* (%). Continuous variables were compared using Mann–Whitney *U*-tests, and categorical variables were compared using the Chi-squared test.

**Table 4:** Diagnostic performance of donor biomarkers for delayed graft function.

Biomarker	AUROC 95% CI	*p* Value	Sensitivity Specificity (%)	Biomarker Cut-Off Values	OR 95% CI	*p* Value
sIL-18 pg/mL	0.706 [0.577–0.835]	0.002	90 46.6	147.3	3.462 [1.665–36.906]	0.003
sKIM-1 pg/mL	0.694 [0.562–0.826]	0.004	95 35.71	161.3	10.0 [1.246–80.245]	0.009
uKIM-1 ng/mL	0.710 [0.574–0.846]	0.002	93.8 53.7	1.2	9.5 [1.158–77.908]	0.014
uNGAL pg/mL	0.717 [0.5914–0.8217]	0.001	87.5 53.1	1161.3	7.913 [1.623–38.352]	0.008

AUROC—area under the receiver-operating characteristic curve; CI—confidence interval; OR—odds ratio; sIL-18—serum interleukin-18; sKIM-1—serum kidney injury molecule-1; uKIM-1—urine kidney injury molecule-1; uNGAL—urine neutrophil gelatinase–associated lipocalin; Pearson Chi-square test.

**Table 5:** Univariate and multivariate logistic regression analyses for predictors of delayed graft function.

Univariate					Multivariate			
	B Coefficient	OR	95% CI	*p* Value	B Coefficient	OR	95% CI	*p* Value
Donor age (year)	0.77	1.080	1.022–1.141	0.006				
Expanded-criteria donors	1.583	4.87	1.567–15.13	0.006	2.204	9.016	2.04–40.23	0.004
Dialysis vintage (months)	0.02	0.002	0.996–1.008	0.571				
Cold ischemia time (hours)	0.193	1.213	1.062–1.385	0.004	0.278	1.320	1.074–1.624	0.008
Donor creatinine (µmol/L)	0.007	1.007	0.993–1.022	0.322				
KDPI, %	0.036	1.037	1.012–1.062	0.004				
Log-transformed sIL-18	2.819	16.765	1.732–162.283	0.015	1.998	0.136	0.022–0.824	0.030
Log-transformed sKIM-1	2.423	11.279	1.802–70.603	0.010				
Log-transformed uKIM-1	2.812	16.645	1.703–162.707	0.016	2.50	12.178	1.226–120.975	0.033
Log-transformed uNGAL	1.127	3.085	0.849–11.209	0.087				

KDPI—kidney donor profile index; sIL-18—serum interleukin-18; sKIM-1—serum kidney injury molecule-1; uKIM-1—urine kidney injury molecule-1; uNGAL—urine neutrophil gelatinase–associated lipocalin; B coefficient—regression coefficient; CI—confidence interval; OR—odds ratio. Biomarker values were log_10_-transformed.

**Table 6:** Univariate and multivariate linear regression analyses predicting 1-year graft functioning.

Univariate				Multivariate		
	β Coefficient	95% CI	*p* Value	β Coefficient	95% CI	*p* Value
Donor age (year)	−0.437	−0.926 to −0.290	*p* < 0.001	−0.50	−1.056 to −0.288	0.01
Donor creatinine	0.062	−0.111 to 0.184	0.624			
Cold ischemia time (hours)	−0.317	−2.742 to −0.375	0.011	−0.298	−3.288 to −0.92	0.039
Dialysis vintage (months)	−0.062	−0.227 to 0.140	0.637			
DGF	−0.304	−24.439 to −2.776	0.015			
Log-transformed sKIM-1	−0.176	−25.5 to 4.739	0.175			
Log-transformed sNGAL	0.263	0.803 to 24.852	0.026			
Log-transformed uNGAL	0.302	0.325 to 22.732	0.044	0.32	1.362–24.084	0.029
Log-transformed uKIM-1	−0.329	−38.88 to −2.66	0.026			

β—standardized beta coefficient; CI—confidence interval; DGF—delayed graft function; KDPI—kidney donor profile index; sKIM-1—serum kidney injury molecule-1; uKIM-1—urine kidney injury molecule-1; NGAL—urine neutrophil gelatinase-associated lipocalin (in serum; in urine).

## Data Availability

The datasets used and/or analyzed during the current study are available from the corresponding author on reasonable request.

## Supplementary Materials

The following supporting information can be downloaded at: https://www.mdpi.com/article/10.3390/diagnostics14070717/s1, Table S1: Results of donor biomarker values; Table S2: Characteristics of recipient outcomes, stratified by delayed graft function.

## Abbreviations

AKI = acute kidney injury, ATG = antithymocyte globulin, AUROC = area under the receiver-operating characteristic curve, CI = confidence interval, CXCL10 = C-X-C motif chemokine 10, DGF = delayed graft function, ECD = expanded-criteria donor, eGFR = estimated glomerular filtration rate, HD = hemodialysis, HLA = human leukocyte antigen, IGF = immediate graft function, KDPI = kidney donor profile index, NGAL = neutrophil gelatinase-associated lipocalin, ROC = receiver-operating characteristic, SCD—standard criteria donors, sIL-18 = serum interleukin-18, sNGAL = serum neutrophil gelatinase-associated lipocalin, sCR = serum creatinine, uIL-18 = urinary interleukin-18, uKIM-1 = urinary kidney injury molecule-1, uKIM-1 = urinary kidney injury molecule-1, uNGAL = urinary neutrophil gelatinase-associated lipocalin.
