# Supplementary material for: Deceased Kidney Donor Biomarkers: Relationship between Delayed Kidney Function and Graft Function Three Years after Transplantation
Source: Diagnostics (Basel). 2024 Mar 28;14(7):717. doi: 10.3390/diagnostics14070717 (PMC11011901; doi:10.3390/diagnostics14070717)
Supplement: Supplementary file 1 [file diagnostics-14-00717-s001.zip › diagnostics-2870844-supplementary.pdf]

Table S1. Baseline characteristics of donors

**Clinicals Characteristics**	**n=43**
Age, years	53.51 (13.33)
Female n (%) / Male n (%)	18 (41.9) / 25 (58.1)
Body mass index, kg/m^2	25.87 (3.62)
Cause of death	
Head trauma, n (%)	6 (14)
Cerebral vascular accident, n (%)	31 (72.1)
Other, n (%)	6 (14)
Terminal serum creatinine, µmol/l	95.13 (39.35)
KDRI	1.1686 (0.358)
KDPI, (%)	59.14 (26.57)
KDPI >85 %	8 (18.5%)
Diuresis ml/24 h	3330 (2400-4000)
Expanded criteria donors, n (%)	23 (53.5)
Donation after circulatory death, n (%)	1 (2.3)
History of hypertension n (%)	25 (58.1)
Use of any inotrope, n (%)	34 (79.1)
Serum NGAL pg/ml	28535.0 (13713.0-54908.4)

Serum IL-18 pg/ml	168.93 (126.8-303.2)
Serum KIM-1 pg/ml	236.4 (160.9-389.3)
Serum CXCL10 pg/ml	41.9 (29.0-108.2)
Urinary NGAL pg/ml	1299.0 (1041.5-5321.2)
Urinary IL-18 pg/ml	12.4 (4.7-17.6)
Urinary KIM-1, ng/ml	2.4 (0.9-4.4)
Urinary CXCL10, ng/ml	29.8 (19.6-52.5)

Results are presented as mean ± SD or n (%), quantitative variables are presented as medians (interquartile ranges). CXCL10 - C-X-C motif chemokine 10, IL-18 - interleukin-18, KDPI - Kidney Donor Profile Index; KDRI - Kidney Donor Risk Index, KIM-1 - kidney injury molecule-1, NGAL-neutrophil gelatinase–associated lipocalin.

Table S2. Characteristics of recipient outcomes, stratified by delayed graft function

	**Total**	**IGF**	**DGF**	***P* value**
eGFR at 1 month, ml/min/1,73m^2	39.70 (15.68)<br>41 (6.4-72)	44.62 (14.3)<br>45.5 (14-72)	27.28 (11.7)<br>27.4 (6.4-47)	**<0.001**
eGFR at 3 months, ml/min/1,73m^2	43.85 (16.47)<br>43.5 (12.7-89)	44.6 (16.2)<br>46.8 (21-89)	36.4 (15.2)<br>37 (12.7-58)	**0.022**
eGFR at 6 months, ml/min/1,73m^2	47.69 (21.94)<br>47 (3-101)	51.4 (22.2)<br>50 (3-101)	37.8 (18.4)<br>37 (10-68)	**0.024**
eGFR 12 months, ml/min/1,73m^2	52.1 (19.12)<br>47.5 (11-99)	55.3 (18.2)<br>51 (23.8-99)	41.7 (18.9)<br>39 (11-72)	**0.024**
eGFR 24 months, ml/min/1,73m^2	53.38 (19.67)<br>50 (17-102)	55.6 (19.8)<br>50 (17-102)	45.3 (17.7)<br>44.7 (18-67)	0.146
eGFR 36 months, ml/min/1,73m^2	55.50 (19.59)<br>54 (20-112.5)	58.4 (18.9)<br>55.45 (29-119.5)	45.1 (19.2)<br>43.2 (20-81.2)	**0.038**
Acute rejection during 1st year, n (%)	8 (10.5)	3 (5.5)	5 (23.8)	**0.033**

eGFR- estimated glomerular filtration rate, IGF - immediate graft function; DGF- delayed graft function.

Values reported are presented as means (SD) and as medians (min.-max.) or n (%).

Kruskal-Wallis tests
